# Risk of malignancy in adrenal tumors in patients with a history of cancer

**DOI:** 10.3389/fonc.2023.1018475

**Published:** 2023-03-27

**Authors:** Radosław Samsel, Karolina Nowak, Lucyna Papierska, Edyta Karpeta, Katarzyna Roszkowska-Purska, Wacław Smiertka, Tomasz Ostrowski, Eryk Chrapowicki, Alan Grabowski, Dorota Leszczyńska, Andrzej Cichocki

**Affiliations:** ^1^ Department of Surgery, Clinic of Surgical Oncology and Neuroendocrine Tumors, Maria Sklodowska-Curie National Research Institute of Oncology, Warsaw, Poland; ^2^ Department of Endocrinology, Medical Centre of Postgraduate Education, Bielański Hospital, Warsaw, Poland; ^3^ Department of Surgical and Transplantation Nursing and Extracorporeal Therapies, Medical University of Warsaw, Warsaw, Poland; ^4^ Department of General and Transplant Surgery , Medical University of Warsaw, Warsaw, Poland; ^5^ Department of Pathology, Maria Sklodowska-Curie National Research Institute of Oncology, Warsaw, Poland

**Keywords:** adrenal tumors, adrenal malignancy, adrenal metastases, adrenalectomy, risk of adrenal malignancy

## Abstract

**Purpose:**

Adrenal gland is a common site of metastasis and on the other hand, metastases are the most frequent malignant adrenal tumors. The aim of this study was to estimate the risk of malignancy in suspicious adrenal mass in patients with a history of cancer.

**Methods:**

This is a single-center retrospective analysis of patients with adrenal tumors treated previously for different types of cancers. Between 2004 and 2021 a hundred and six such patients were identified. Mean age of patients was 62.6 years (30-78), and mean time from oncologic treatment was 55.8 months (0-274). The most common primary cancer was kidney (RCC): 29 (27.4%), colon/rectum (CRC): 20 (18.9%) and lung (NSCLC): 20 (18.9%).

**Results:**

Of 106 patients, 12 had hormonally active (HA) (11,3%) and 94 (88,7%) non active (HNA) tumors In group of patients with HA tumours 4 had hypercortisolaemia and 8 had elevation of urinary metanephrines. In the first group of HA patients pathology confirmed preoperative diagnosis of adrenocortical cancer and no metastasis was found. In all patients from the second group pheochromocytomas were confirmed. Primary (PM) and secondary (SM) malignancies were found in 50 patients (47.2%). In hormone inactive group only SM - 46/94 (48.9%) were diagnosed. The odds that adrenal lesion was a metastasis were higher if primary cancer was RCC (OR 4.29) and NSCLC (OR 12.3). Metastases were also more likely with high native tumor density, and bigger size in CT. The cut-off values for tumor size and native density calculated from receiver operating characteristic (ROC) curves were 37mm and 24, respectively.

**Conclusion:**

Risk of malignancy of adrenal mass in a patient with a history of cancer is high (47,2%), regardless of hormonal status. 47,2% risk of malignancy. In preoperative assessment type of primary cancer, adrenal tumour size and native density on CT should be taken into consideration as predictive factors of malignancy. Native density exceeding 24 HU was the strongest risk factor of adrenal malignancy (RR 3.23), followed by history of lung or renal cancer (RR 2.82) and maximum tumor diameter over 37 mm (RR 2.14).

## Introduction

Incidentally detected adrenal masses in patients with no known malignancy occur in 1-5% of all abdominal computed tomography (CT) examinations (1, 2), and prevalence of such findings increases with age (3, 4). Majority of adrenal masses in patients with no known malignancy have been shown to be benign (1). In the absence of primary malignancy the risk of the adrenal lesion to be malignant is approximately one in a thousand (5).

Adrenal gland is one of the most prevalent sites for metastases from various malignant tumors. The most common type of adrenal tumors are benign cortical adenomas (6), however metastases are the most frequent malignant tumors of the adrenal gland and second cause of an adrenal mass (7). Presence of adrenal metastases in patients with a history of cancer varies in different series between 10 and 27% (8, 9). Rich sinusoidal blood supply of adrenal glands may favor this localization (9). Adrenal glands are the fourth most common site of metastases in malignant diseases (10).

Surveillance protocols in cancer treated patients have increased identification of incidental adrenal lesions. Good proportion of them are metastases. Sensitive and reliable methods of diagnostic imaging lead to earlier detection of adrenal metastases. In majority of cases it’s a manifestation of disseminated disease, however detection and surgical resection of isolated adrenal metastasis can improve prognosis in selected patients (11, 12). When adrenal mass is found in a patient with a history of extra-adrenal malignancy it is important to try to distinguish primary adrenal tumor from metastasis. Determination of malignant potential is usually a function of radiographic characteristics and size of an adrenal mass.

The aim of the study was to assess the risk of malignancy in adrenal tumors found in patients with a history of treatment for primary extra-adrenal cancer.

Retrospective chart review of all patients with a history of malignancy who underwent resection of an adrenal mass was performed.

## Patients and methods

Medical records of patients referred to our center for adrenal mass with a history of cancer were reviewed. Pre-operative clinical, radiological and biochemical data as well as detailed operation and histology reports retrieved. Radiology and pathology reports were intentionally not re-evaluated, to keep the data entirely observational. All patients were initially evaluated and followed outside our hospital with adrenal CT protocol. Native, enhanced (after 1 min) and delayed (after 15 min) density was measured and an absolute contrast washout and relative contrast washout were calculated according to standard methods. Adrenal mass size was defined as the largest diameter on axial plane CT, attenuation on unenhanced CT was given in Hounsfield units (HU) and measured as a region of interest (ROI). A ROI was placed at the center of the lesion and its size and shape were adjusted to avoid calcification, cystic or necrotic area. Neither MRI nor PET-scan were used in the routine decision tree for the study purpose.

From January 2004 till December 2021, six hundred thirty patients with various adrenal tumors were referred to our center for surgical treatment. Initial diagnostic procedures (hormonal status, imaging) were most often performed in referring centers. As for functional status, at least levels of urinary catecholamines and plasma cortisol level with dexamethasone suppression test were done in all patients as well as others routine hormonal tests.

According to current guidelines, adrenal biopsies were not performed. Indications for surgery were determined on clinical history, cross-sectional imaging and/or hormonal activity. Of these, a hundred and six (16,8%) patients had a history of treatment for solid cancer. Patients with a history of hematological malignancies (n=2) were not included in this analysis. At adrenalectomy, the disease was clinically and radiographically limited to the adrenal gland in all patients. Extra-adrenal malignancy in every case was excluded with abdominal, pelvic and chest CT.

General anesthesia and appropriate antibiotic prophylaxis with second generation cephalosporin (cefuroxime) were used according to hospital protocol. All patients received low molecular weight heparins perioperatively as venous thromboembolism prophylaxis. Student’s t test was used to analyze variables with normal distribution. In the presence of non-normally distributed data, nonparametric tests (Mann-Whitney U test/χ^2^ test) were used. A multivariate logistic regression was used to identify predictive factors for metastasis in patients with a history of extra-adrenal malignancy having solitary adrenal mass. A receiver-operating characteristic (ROC) curve was used to identify the cut-off value of size, precontrast density and absolute contrast washout between adrenal metastases and benign lesions on preoperative CT. Analysis was performed with STATISTICA 13.3 software (TIBCO Software Inc, Tulsa, Oklahoma, United States). *P* value less than.05 was considered significant.

## Results

**Figure 1:**
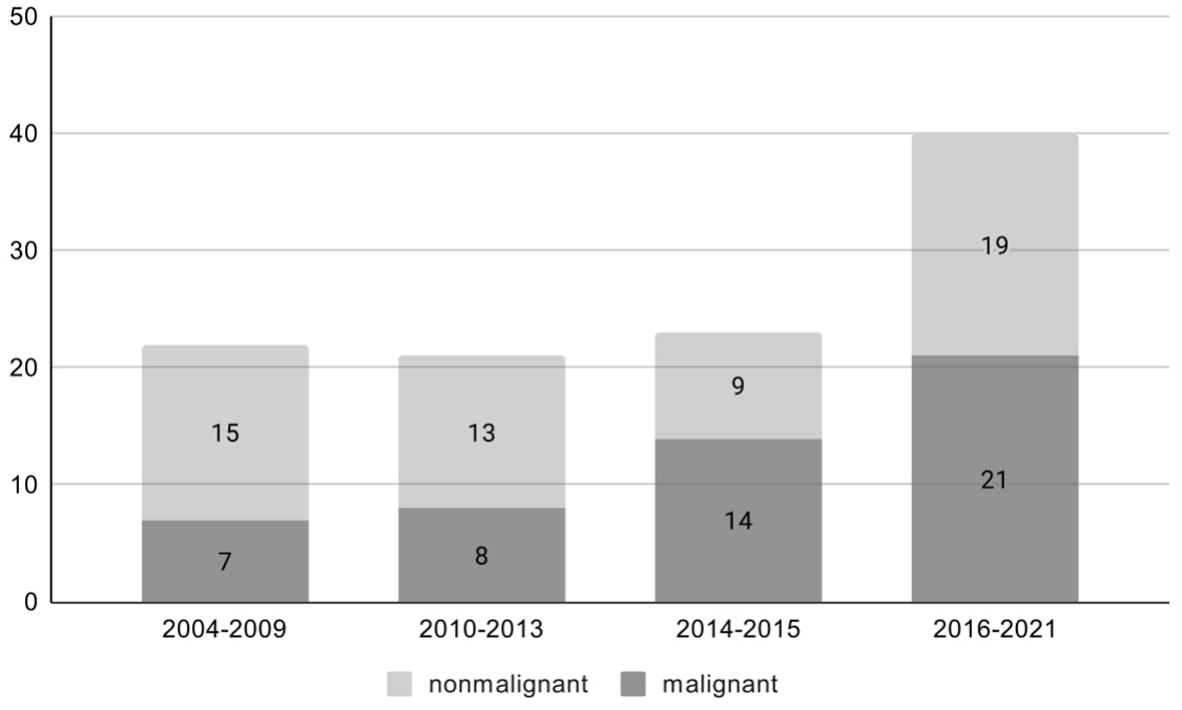
Percentage of malignant lesions over time (p=0.17).

There were 106 patients with an adrenal mass in the setting of a prior or concurrent diagnosis of at least one extra-adrenal malignancy who were treated with adrenalectomy. The number of treated patients increased over time, accompanied by not significantly higher number of malignant lesions (p=0.17) **Figure 1**. Sixty-one patients (57,5%) were women. Mean age at the time of operation was 62.6 ± 9.4 years (range 30-78), and it was similar for men 62.4 ± 9.2 and women 62.4 ± 9.6. Baseline characteristics of the study population are presented in **Table 1**.

**Table 1:** Baseline characteristic of the study population.

Characteristic	Non-missing	N (%) or mean (SD)
Total number of participants	106	
Sex, W/M	106	61(57.55%)/45(42.45%)
Age (years)	104	62.62 (SD ± 9.4) range 30-78
Age, F (years)	60	62.43 (SD ± 9.65) range 30-78
Age, M (years)	44	62.43 (SD ± 9.17) range 36-78
Hormonal activity	106	12 (11.32%)
Tumour size (mm)	89	44.2 (SD ± 28) range 11-134
Native density in CT scan	48	25.2 (SD ± 11.5) range 0-45
Enhanced density in CT scan (1 min after contrast administration)	41	71.3 (SD ± 38) range 15-200
Delayed density in CT scan (10 min after contrast administration)	33	56.5 (SD ± 19.3) range 15-100
Absolute contrast washout (%)	33	40.1 (SD ± 22.2) range 3.3-100
Relative contrast washout (%)	33	24 (SD ± 16.6) range 2-81.25
Time from primary cancer to adrenalectomy (months)	103	54.9 (SD ± 61.4) range 0-274.3

In a hundred and six patients, there were 112 cancers diagnosed: 4 patients were diagnosed with 2 cancers (RCC+NSCLC, RCC+melanoma, CRC+breast, CRC+ NSCLC) and one patient had history of 3 different cancers (RCC, thymoma and prostate). Four patients with multiple cancer diagnosis had adrenal metastases and 1 patient with rectal and breast cancer (16 and 6 years prior to adrenalectomy, respectively) was diagnosed with 50 mm non-functioning adenoma. Detailed characteristics of patients with single cancer diagnosis are presented in **Table 2**.

**Table 2:** characteristic of malignancies treated before adrenalectomy (N=101).

Type of cancer	Number (%)	M/F	Age (years) Mean (± SD) [range]	Time from primary cancer to adrenalectomy (months) mean (± SD) [range]
RCC	26 (25.74%)	14/12	61.4 (SD ± 5) [53-77]	66.6 (SD ± 54.2) [6.8-184.8]
NSCLC	18 (17.82%)	8/10	59.7 (SD ± 7.3) [47-73]	21.1 (SD ± 23) [0-87.5]
CRC	18 (17.82%)	11/7	66.4 (SD ± 10.4) [36-78]	25.2 (SD ± 29.7) [0-124.3]
Breast	13 (12.87%)	0/13	64.2 (SD ± 8.6) [51-77]	96.7 (SD ± 87.7) [8.5-274]
Prostate	5 (4.95%)	5/0	73 (SD ± 3.9) [67-77]	84.7 (SD ± 62.2) [20.7-179]
Utreus	4 (3.96%)	0/4	63 (SD ± 13.2) [48-73]	88.8 (SD ± 102.5) [0-233]
Thyroid	4 (3.96%)	3/1	50.8 (SD ± 5) [44-56]	105.5 (SD ± 124) [6.3-264]
Ovary	3 (2.97%)	0/3	69 (SD ± 8.2) [62-78]	69.4 (SD ± 84.5) [20.3-167]
Other diagnosis*	10 (9.9%)	1/9	55.4 (SD ± 13.5) [30-74]	32 (SD ± 28.9) [7.1-88.9]

M, male; F, female; RCC, Renal Cell Cancer; CRC, Colorectal Cancer; NSCLC Non Small Cell Lung Cancer. *Other: malignant melanoma-1, thymoma-1, soft tissue sarcoma – 2, neuroendocrine cancer- 2, stomach- 1, bladder – 1, palnoepithelial ani – 1, cervix – 1.

Fifty-seven left, 41 right and 6 bilateral adrenalectomies were performed. In two patients tumors were unresectable and only biopsy was carried out. Ninety open and 16 laparoscopic procedures were executed. Typical surgical approach was transabdominal lateral flank incision. Laparoscopic technique was introduced in our center in 2013, and since then majority of adrenalectomies have been performed with transabdominal approach. There were no fatalities or major complications associated with surgery. Three patients (2.8%) had mild complications, grade I and II in the Clavien-Dindo Classification (1 postoperative pneumonia, 1 wound hematoma without the need for surgical intervention, and 1 prolonged postoperative pain). Average postoperative hospital stay was 6.5 days (range 2-30). One prolonged hospitalization (30 days) was related to severe wound pain requiring prolonged opioids.

Primary or secondary malignancy was found in 50 of 106 (47.2%) patients. Risk of primary malignancy was relatively low, 4/106 (3,8%). There were 12 patients (11.3%) with hormonally active (HA) tumours of whom 4 had elevated cortisol levels unresponsive to dexamethasone suppression and 8 had elevations of urinary catecholamine levels. None of the patients had hyperaldosteronism or other type of hormonal abnormalities. In the first group of HA patients pathology confirmed preoperative diagnosis of adrenocortical cancer and no metastasis was found. In all patients from the second group pheochromocytoma was confirmed. Adrenocortical carcinoma was recognized only in females, after treatment of breast (n=2) and endometrial (n=2) cancers. In hormonally nonactive tumor group (HNA, n=94) all malignancies proved to be metastases (n=46, 48.9%). In HNA patients with benign lesions histopathological examination revealed 33 adenomas, 8 ACTH-independent macronodular adrenal hyperplasia (AIMAH), 3 post-hemorrhage changes, 2 cystic lesions and 2 cases of pheochromocytoma. Tumor characteristic in patients without hormonal activity is presented in **Table 3**.

**Table 3:** Tumour characteristic in patients without hormonal activity.

Characteristic	Benign lesions (N=48)	Metastases (N=46)	p value
Sex, F/M	35/13	18/28	**0.001**
Age (years), mean ± S.D.	62.7 ± 9.9	63.8 ± 6.7	0.7
Time from primary cancer to adrenalectomy (months), mean ± S.D. median (25th – 75th pctl) range	65.8 ± 75.25 28.4 (15.3-80.9) [0-274]	47.8 ± 48 29 (14.7-59) [2-184]	0.7
Tumour size (mm), mean ± S.D. median (25th – 75th pctl) range	34 ± 20.5 29 (23.5-36.5) [14-134]	48 ± 28.6 43 (27-64) [11-123]	**0.01**
Native density in CT scan (HU) mean ± S.D. median (25th – 75th pctl) range	19.4 ± 13 17 (11.5-27) [0-42]	30.4 ± 7.5 28 (31-34) [16-45]	**0.002**
Enhanced density in CT scan (HU) mean ± S.D. median (25th – 75th pctl) range	60 ± 47 53 (20-80) [15-200]	76 ± 27.5 68 (55-102) [35-130]	0.08
Delayed density in CT scan (HU) mean ± S.D. median (25th – 75th pctl) range	51 ± 24 55 (40-64) [15-88]	59 ± 18 54 (48-70) [30-100]	0.3
Absolute contrast washout (%) mean ± S.D. median (25th – 75th pctl) range	50 ± 31 43 (29-48) [16-100]	34 ± 16 31 (21-50) [3-71]	0.17
Relative contrast washout (%) mean ± S.D. median (25th – 75th pctl) range	27 ± 22 24 (20-26) [5.5-81.25]	20 ± 12 18 (12-25) [2-51]	0.35

Bold values are statistically significant.

In multivariable regression analyses of potential risk factors for metastases before adrenalectomy, tumor size, native density, and primary RCC and NSCLC were significant predictive factors for adrenal metastasis – **Tables 4**, **5**.

**Table 4:** Multivariable regression analysis of potential risk factors for metastasis before adrenalectomy (RCC included as a covariate).

			Univariate			Multivariate		
Variable	N of 94	N+	OR	95% CI	P	OR	95% CI	P
Sex, Male	94	45	4.2	1.76-10	**0.001**	3.5	0.56-21.65	0.18
Tumour size (mm)	79		1.03	1.0-1.05	**0.02**	1.04	1.0-1.08	**0.03**
Native density in CT scan (HU)	45		1.11	1.03-1.18	**0.004**	1.12	1.02-1.22	**0.014**
RCC	94	29	5.37	1.98 – 14.4	**0.001**	4.29	0.68-26.8	0.12

N+ - number of patients who met the given criteria. Forward stepwise selection at 0,1 significance level was used for variable selection in multivariable model.

In multivariable model (χ2 = 22.87, p=0.0001) tumour size, native density and RCC diagnosis were significant predictive factors for adrenal metastasis.

Bold values are statistically significant.

**Table 5:** Multivariable analysis of potential risk factors for metastasis before adrenalectomy with NSCLC diagnosis as a covariate.

			Univariate			Multivariate		
Variable	N of 94	N+	OR	95% CI	P	OR	95% CI	P
Sex, Male	94	45	4.2	1.76-10	**0.001**	11.3	1.04-121.8	0.18
Tumour size (mm)	79		1.03	1.0-1.05	**0.02**	1.06	1.01-1.11	**0.02**
Native density in CT scan (HU)	45		1.11	1.03-1.18	**0.004**	1.15	1.03-1.29	**0.016**
NSCLC	94	20	3.06	1.06-8.85	**0.04**	12.3	1.08-139.2	**0.04**

N+ - number of patients who met the given criteria. Forward stepwise selection at 0,1 significance level was used for variable selection in multivariable model.

In multivariable model (χ2 = 25.73, p=0.0000) tumour size, native density and NSCLC diagnosis were significant predictive factors for adrenal metastasis.

Bold values are statistically significant.

Cut-off values for tumor size and native density were calculated from receiver operating characteristic (ROC) curves. Risk of malignancy is higher for tumor exceeding 37 mm (59% sensitivity and 77.5% specificity) and with native density over 24 HU (80% sensitivity and 75% specificity). I the group of 34 patients with together diameter exceeding 37 mm and native density over 24 HU, 23 had malignant lesions on pathologic report (67,6%) In the opposite of 13 patients with tumors 37mm and smaller and native density lower than 24 HU only 1 patients finally had malignant adrenal tumor (7,8%).

Risk factor was calculated and was the strongest for native density exceeding 24 HU (RR 3.23),followed by history of lung or renal cancer (RR 2.82) and maximum tumor diameter over 37 mm (RR 2.14) **Table 6**.

**Table 6:** Relative risk of adrenal malignancy was calculated for identified risk factors.

Relative risk of lesion to be malignant			
	95% CI	RR	p-value
Tumor size >37mm	1.315, 3.491	2.142857	0.001106
Primary lung or renal cancer	1.765, 4.505	2.819444	0.000007
Native density >24 HU	1.461, 7.144	3.230769	0.001887

## Discussion

A solitary adrenal mass is identified at the time of cancer diagnosis or during follow-up in many oncological patients. Few reports have addressed an issue of evaluation and treatment of adrenal masses in patients with a history of malignancy. Studies of adrenal incidentaloma usually exclude patients with synchronous or metachronous extra-adrenal malignancies (13) whereas reports of adrenalectomy for metastatic cancer exclude those with primary adrenal tumors (14, 15). Patients with adrenal mass and a history of malignancy constitute an important clinical problem. In patients with disseminated metastatic disease diagnosis of adrenal mass rarely alters treatment decision making. However, if an adrenal lesion is a unique sign of metastatic spread, it may have an essential impact on patient management.

Adrenalectomy is indicated for hormonally active tumors or tumors in which a benefit from potential malignancy treatment outweighs the risk of surgery, including treatment for isolated metastases from solid-organ cancers. Majority of adrenalectomies (81,5%), are performed for benign disease, mostly for pheochromocytoma followed by Cushing’s syndrome, Conn’s syndrome, and non-functioning adrenal adenoma. In malignancies, surgery is most often done for metastases, adrenocortical carcinoma and malignant pheochromocytoma (16). This seems more often to be the case in patients with a history of or synchronous malignancy (2). In an adrenal mass exceeding 4 cm, that grows in one-year follow up imaging, probability of metastatic character is up to 71% (17). In our series total risk of malignancy was 47.2%: 48.9% in patients without hormonal activity and 33.3% in hormonally active group.

Adrenocortical cancers occurred in women with a history of breast and endometrial cancers. Such coincidence may result from genetic mutations which may be found in Li-Fraumeni and Lynch syndromes (18, 19).

Most common primary cancers which metastasized to adrenals in our study were RCC (27.4%), CRC (18.9%) and NSCLC (18.9%) and this proportions are similar to previously reported. The most common primary sites reported are lung cancer (32%), RCC (22%) and melanoma (15%) (20). In another large series, most frequent primary tumors were lung (46,6%), colorectal (13,5%), and RCC (11,7%) (11). There are also reports with high prevalence of GI cancers (21).

Decision regarding surgery is usually based on diagnostic imaging. Risk of malignancy is determined by tumor size, radiographic features such as irregular margins, high density, slow contrast washout, presence of necrosis, area of hemorrhage and calcifications. CT is considered the most useful imaging technique in differential diagnosis of adrenal masses (22). Measurement of Hounsfield units (HU) in an unenhanced CT is very useful in discriminating benign from malignant mass. Recommended threshold for CT density of benign lipid-rich adenomas correctly is <=10 HU. At this threshold, pooled sensitivity and specificity were 71% and 98% respectively (23). Lipid-poor adenomas with attenuation value of less than 10 HU represent 10-40% of all adenomas (24). None of the metastatic tumors we found had native density lower than 10 HU. Characterization of adrenal mass with contrast-enhanced CT takes an advantage of specific perfusion pattern of different tumors. Malignant lesions including metastases enhance rapidly but demonstrate a slower washout of contrast medium (25). An absolute contrast washout of >60% and a relative contrast washout of >40% characterize an adenoma with sensitivity and specificity of 98 and 92% respectively (26, 27). It is worth to mention that more recent studies revealed that washout CT may be insufficient to reliably diagnose adrenal masses (28). Dedicated adrenal imaging including CT attenuation measurements with washout characteristic is highly recommended in patient with adrenal tumours previously treated for extra-adrenal malignancy (29). High precontrast HU (>36), and presence of metachronous mass should rise alert of metastasis in patients with extra-adrenal cancer who present with solitary adrenal mass (30). Our data revealed lower native density value cutoff (24 HU), above which risk of metastasis was statistically higher. It has been proven that adrenal tumour size is highly connected to malignancy. In unselected groups of patients with and without previous oncologic treatment, risk of malignancy increased with tumour size to 37.7% when lesion exceeded 6 cm (31). It had been suggested that an adrenal mass > 22 mm in patients with extra-adrenal cancer may be indicative of metastasis (sensitivity 73.1%; specificity 78.5%) (32). Our results confirm that adrenal mass bigger than 37 mm was related to higher risk of malignancy. This observation is in line with a recent study where tumor size > 3.2 cm, and features of malignancy on imaging were independent predictive factors for adrenal malignancy (33). Nevertheless in small and ambiguous tumors, CT may be insufficient to establish proper diagnosis and Positron Emission Tomography and Computed Tomography (PET-CT) may be necessary, although also has limitations. Many different benign adrenal lesions can show increased FDG uptake (34).

There were 11.3% of functional tumours in the study group. Regardless of concurrent or a history of malignancy, all patients with an adrenal mass should undergo standard hormonal screening to identify functional neoplasm, at least to carry out tests to exclude pheochromocytoma (formerly daily urinary metanephrines, nowadays mainly free plasma metanephrines), as it is then necessary to use alpha-blockade in preparation for surgery. However, limitations of the screening must be recognized. False-negative urinary test results in patients with final histopathological diagnosis of pheochromocytoma are common (35, 36). We identified 2 patients (2,1%) who had biochemically silent pheochromocytoma, which is significantly less than 6.57% as previously reported (37).

Results of surgical treatment for selected patients with isolated adrenal metastases of different types of cancers are satisfactory (38, 39). Resection of adrenal metastases in carefully chosen patients seems reasonable. There were no mortalities and only minor morbidity related to adrenalectomy in our series. Adrenalectomy is an effective treatment option in patients with good control of extra-adrenal disease, acceptable performance status and absence of significant comorbidities. We concur that all patients with a history of malignant disease and newly diagnosed adrenal lesion should be suspected for metastases and referred to surgical treatment with no delay (40, 41). Follow-up rules used for incidentalomas (42, 43) must not be applied to these cases since unnecessary delay may affect patient’s life expectancy. Factors such as sex, type of primary cancer, tumour size and CT characteristics may identify patients in whom treatment should be performed quickly.

## Conclusions

In summary, analysis of this comprehensive series of patients with a history of extra-adrenal malignancy operated for adrenal tumours showed 47,2% risk of malignancy. In preoperative assessment type of primary cancer, adrenal tumour size and native density on CT should be taken into consideration as predictive factors of malignancy. Native density exceeding 24 HU was the strongest risk factor of adrenal malignancy (RR 3.23), followed by history of lung or renal cancer (RR 2.82) and maximum tumor diameter over 37 mm (RR 2.14).

## Data availability statement

The raw data supporting the conclusions of this article will be made available by the authors, without undue reservation.

## Ethics statement

Authors have obtained ethical approval from an ethics review board of Medical Centre of Postgraduate Education (30/2022), to perform and publish this study.

## Author contributions

RS - Study conception and design, acquisition of data, analysis and interpretation of data, drafting of manuscript. KN - Study conception and design, acquisition of data, analysis and interpretation of data, critical revision of manuscript. LP - Study conception and design, acquisition of data, analysis and interpretation of data, critical revision of manuscript. EK - Analysis and interpretation of data, critical revision of manuscript. KR-P - acquisition of data, drafting of manuscript, critical revision of manuscript. WS - acquisition of data, drafting of manuscript, critical revision of manuscript. TO - acquisition of data, analysis and interpretation of data, critical revision of manuscript. EC - acquisition of data, drafting of manuscript, critical revision of manuscript. AG - acquisition of data, analysis and interpretation of data, critical revision of manuscript. DL - acquisition of data, analysis and interpretation of data, critical revision of manuscript. AC - Study conception and design, acquisition of data, drafting of manuscript, critical revision of manuscript. All authors contributed to the article and approved the submitted version.
